# Fibrosis-4 index efficiently predicts chronic hepatitis and liver cirrhosis development based on a large-scale data of general population in Japan

**DOI:** 10.1038/s41598-022-24910-2

**Published:** 2022-11-27

**Authors:** Nobutake Yamamichi, Takeshi Shimamoto, Kazuya Okushin, Takako Nishikawa, Hirotaka Matsuzaki, Seiichi Yakabi, Mami Takahashi, Ryoichi Wada, Kazuhiko Koike, Mitsuhiro Fujishiro

**Affiliations:** 1 Center for Epidemiology and Preventive Medicine, The University of Tokyo Hospital, 7-3-1, Hongo, Bunkyo-ku, Tokyo, Zip Code: 113-8655 Japan; 2 Department of Gastroenterology, Graduate School of Medicine, The University of Tokyo, 7-3-1, Hongo, Bunkyo-ku, Tokyo, Japan; 3 Kameda Medical Center Makuhari, CD-2, 1-3, Nakase, Mihama-ku, Chiba-City, Japan; 4 Kanto Central Hospital, 6-25-1, Kamiyouga, Setagaya-ku, Tokyo, Japan

**Keywords:** Disease prevention, Prognosis, Epidemiology, Non-alcoholic fatty liver disease

## Abstract

A non-invasive method to evaluate the fibrosis stage and the risk stratification of non-alcoholic fatty liver disease (NAFLD) is required. A total of 416,066 generally healthy subjects who underwent health check-ups between 1990 and 2019 were investigated. Fatty liver prevalence greatly increased from the 1990s (21.9%) to the 2000s (37.1%) but showed no considerable change between 2001–2010 (39.2%) and 2011–2019 (35.5%). During the 30 years, the rate of high FIB-4 index (≥2.67) and mean body mass index (BMI) did not markedly change. Fatty liver was significantly associated with BMI, but not with alcohol intake or FIB-4 index. Cox regression analyses for development of chronic hepatitis or liver cirrhosis identified that the risk of developing chronic hepatitis and liver cirrhosis was higher in subjects without fatty liver than in those with it (hazard ratio [HR]=0.09; 95% confidence interval [CI], 0.03–0.22, *p* <0.001 and HR=0.04; 95% CI, 0.01–0.26, *p* =0.001, respectively), and much larger in subjects with a high FIB-4 index (≥ 2.67) than in those without it (HR=78.6; 95% CI, 29.0–213.1, *p* <0.001 and HR=5950.7; 95% CI,761.7–46,491.4, *p* <0.001, respectively). Adjusted survival curves for Cox proportional hazards regression further reinforced these results. In conclusion, the FIB-4 index is a useful indicator of chronic hepatitis and liver cirrhosis development in the general population.

## Introduction

Non-alcoholic fatty liver disease (NAFLD) is the most prevalent chronic liver disease worldwide^1–4^. NAFLD has a wide spectrum; more severe cases are referred to as nonalcoholic steatohepatitis, which is progressive and is related to cirrhosis, hepatocellular carcinoma, and systemic complications, including cardiovascular diseases^5^.

Recently, the fibrosis stage was identified as the most important prognostic factor for liver-related and overall mortality in NAFLD^6,7^. The gold standard for determining the fibrosis stage is liver biopsy, which is invasive and carries a risk of complications^8^. To compensate for the limitations of liver biopsy, several non-invasive tools to evaluate the fibrosis stage of NAFLD have been proposed and validated^9,10^.

The identification of NAFLD patients with advanced fibrosis in a general population setting has also been discussed. Several scoring systems have been evaluated; however, the usefulness of these scores remains debatable^11,12^.

Of the various scoring systems, the Fibrosis-4 (FIB-4) index, which was originally developed for staging liver disease in patients co-infected with human immunodeficiency virus and hepatitis C virus, is the most widely accepted and well-validated^13,14^. As the FIB-4 index is calculated using age, aspartate aminotransferase and alanine aminotransferase levels, and platelet count, it can be applied to past data in an objective manner^14^. The applicability of the FIB-4 index in predicting the future development of liver disorders is the next challenge^15,16^.

Herein, we clarified the time trend of the FIB-4 index and prevalence of fatty liver for 30 years, along with data on alcohol intake and body mass index (BMI) using large-scale cohort data from 1990 to 2019. We further examined the ability of the FIB-4 index to identify future development of significant liver disorders, including chronic hepatitis and cirrhosis, in a large population-based cohort over a long follow-up period.

## Results

### Study subjects

**Figure 1:**
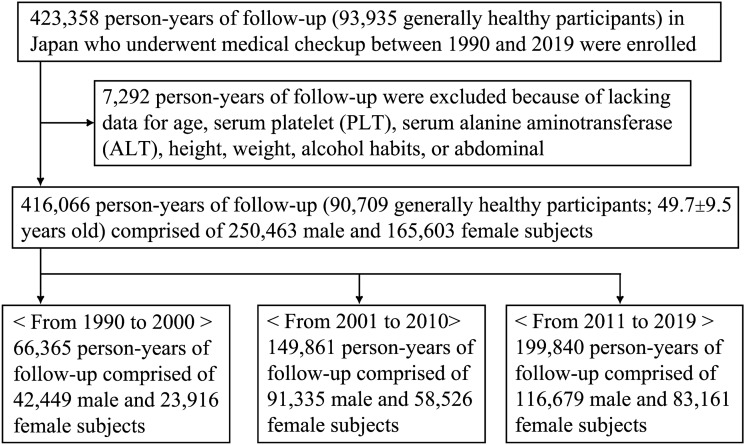
Flowchart of participant recruitment in the present study.

After excluding subjects lacking essential data (Fig. 1), a consecutive 416,066 person-years of follow-up (90,709 generally healthy participants) from 1990 to 2019 were analyzed (mean age, 49.7 ± 9.5 years; range 20–93 years). The average number of follow-ups was 5.5 ± 4.8 and the average duration of follow-ups was 4.8 ± 6.1 years. The study consisted of 66,365 subjects (42,449 men and 23,916 women) between 1990–2000, 149,861 subjects (91,335 men and 58,526 women) between 2001–2010, and 199,840 subjects (116,679 men and 83,161 women) between 2011–2019. The data on fatty liver, FIB-4 index, BMI, and alcohol intake of each age group are shown in Table 1 (total 416,066 subjects), Supplementary Table S1 (250,463 men), and Supplementary Table S2 (165,603 women).

**Table 1:** Detailed data on the prevalence of fatty liver, fibrosis-4 (FIB-4) index, body mass index (BMI), and alcohol intake for all the study subjects who were categorized into five age groups and three periods (1990–2000, 2001–2010, and 2011–2019).

Year	Age group (years)	N	Fatty liver		FIB-4 Index		BMI				Alcohol intake		
			Present	Absent	2.67	< 2.67	< 18.5	≥ 18.5, < 25	≥ 25, < 30	≥ 30	None-Light	Moderate	Heavy
1990–2000	< 40	13,168	2453 (18.6)	10,715 (81.4)	9 (0.1)	13,159 (99.9)	836 (6.3)	9543 (72.5)	2462 (18.7)	327 (2.5)	4354 (33.1)	7444 (56.5)	1370 (10.4)
	40–49	30,959	6818 (22.0)	24,141 (78.0)	77 (0.2)	30,882 (99.8)	1146 (3.7)	22,472 (72.6)	6737 (21.8)	604 (2.0)	11,041 (35.7)	16,236 (52.4)	3682 (11.9)
	50–59	17,610	4139 (23.5)	13,471 (76.5)	263 (1.5)	17,347 (98.5)	523 (3.0)	12,667 (71.9)	4133 (23.5)	287 (1.6)	6300 (35.8)	8689 (49.3)	2621 (14.9)
	60–69	4189	1019 (24.3)	3170 (75.7)	186 (4.4)	4003 (95.6)	159 (3.8)	2982 (71.2)	1000 (23.9)	48 (1.1)	1494 (35.7)	1944 (46.4)	751 (17.9)
	70 <	439	79 (18.0)	360 (82.0)	82 (18.7)	357 (81.3)	37 (8.4)	289 (65.8)	112 (25.5)	1 (0.2)	169 (38.5)	181 (41.2)	89 (20.3)
Total		66,365 (0.0)	14,508 (21.9)	51,857 (78.1)	617 (0.9)	65,748 (99.1)	2701 (4.1)	47,953 (72.3)	14,444 (21.8)	1267 (1.9)	23,358 (35.2)	34,494 (52.0)	8513 (12.8)
2001–2010	< 40	25,500	7350 (28.8)	18,150 (71.2)	6 (0.0)	25,494 (100.0)	2718 (10.7)	17,813 (69.9)	4220 (16.5)	749 (2.9)	9561 (37.5)	13,757 (53.9)	2182 (8.6)
	40–49	54,474	20,727 (38.0)	33,747 (62.0)	67 (0.1)	54,407 (99.9)	3298 (6.1)	37,855 (69.5)	11,426 (21.0)	1895 (3.5)	20,483 (37.6)	28,012 (51.4)	5979 (11.0)
	50–59	53,478	24,005 (44.9)	29,473 (55.1)	340 (0.6)	53,138 (99.4)	2318 (4.3)	37,725 (70.5)	12,258 (22.9)	1177 (2.2)	20,318 (38.0)	25,926 (48.5)	7234 (13.5)
	60–69	14,659	6106 (41.7)	8553 (58.3)	299 (2.0)	14,360 (98.0)	662 (4.5)	10,855 (74.1)	2944 (20.1)	198 (1.4)	5627 (38.4)	6709 (45.8)	2323 (15.8)
	70 <	1750	610 (34.9)	1140 (65.1)	197 (11.3)	1553 (88.7)	123 (7.0)	1283 (73.3)	316 (18.1)	28 (1.6)	722 (41.3)	711 (40.6)	317 (18.1)
Total		149,861 (0.0)	58,798 (39.2)	91,063 (60.8)	909 (0.6)	148,952 (99.4)	9119 (6.1)	105,531 (70.4)	31,164 (20.8)	4047 (2.7)	56,711 (37.8)	75,115 (50.1)	18,035 (12.0)
2011–2019	< 40	21,129	4615 (21.8)	16,514 (78.2)	18 (0.1)	21,111 (99.9)	2474 (11.7)	14,636 (69.3)	3292 (15.6)	727 (3.4)	8020 (38.0)	11,585 (54.8)	1524 (7.2)
	40–49	64,277	20,450 (31.8)	43,827 (68.2)	111 (0.2)	64,166 (99.8)	4916 (7.6)	43,477 (67.6)	13,075 (20.3)	2809 (4.4)	23,291 (36.2)	32,949 (51.3)	8037 (12.5)
	50–59	72,148	29,037 (40.2)	43,111 (59.8)	534 (0.7)	71,614 (99.3)	4284 (5.9)	48,448 (67.2)	16,458 (22.8)	2958 (4.1)	25,041 (34.7)	35,785 (49.6)	11,322 (15.7)
	60–69	35,126	14,387 (41.0)	20,739 (59.0)	847 (2.4)	34,279 (97.6)	2024 (5.8)	24,232 (69.0)	7933 (22.6)	937 (2.7)	13,102 (37.3)	15,749 (44.8)	6275 (17.9)
	70 <	7160	2446 (34.2)	4714 (65.8)	786 (11.0)	6374 (89.0)	485 (6.8)	5073 (70.9)	1459 (20.4)	143 (2.0)	3144 (43.9)	2755 (38.5)	1261 (17.6)
Total		199,840 (0.0)	70,935 (35.5)	128,905 (64.5)	2296 (1.1)	197,544 (98.9)	14,183 (7.1)	135,866 (68.0)	42,217 (21.1)	7574 (3.8)	72,598 (36.3)	98,823 (49.5)	28,419 (14.2)

### Thirty-year trends of fatty liver, FIB-4 index, and body mass index in Japan

As shown in Table 1, compared with that in the 1990s (approximately 22%), the prevalence of fatty liver greatly increased in the 2000s (more than 35%); however, there was no remarkable change between 2001–2010 (39.2%) and 2011–2019 (35.5%). On the other hand, the rate of high FIB-4 index (≥ 2.67) did not markedly change during the 30 years (0.9% in 1990–2000, 0.6% in 2001–2010, and 1.1% in 2011–2019). Further, although BMI did not change notably (mean BMI: 23.1 in 1990–2000, 23.0 in 2001–2010, and 23.1 in 2011–2019), the prevalence of obesity (BMI ≥ 30) continuously increased from 1990 to 2019 (1.9% in 1990–2000, 2.7% in 2001–2010, and 3.8% in 2011–2019).

Although similar trends were observed for both men (Supplementary Table S1) and women (Supplementary Table S2), there was a considerable difference in their prevalence between sexes. The rates of fatty liver, high FIB-4 index (≥ 2.67), and obesity in men were higher than those in women in all three periods (1990–2000, 2001–2010, and 2011–2019) and all age groups (< 40, 40–49, 50–59, 60–69, and > 70 years). In all male and female subjects during the 30 years, the rates of fatty liver were 42.9% and 22.2%, rates of high FIB-4 index (≥ 2.67) were 1.1% and 0.6%, rates of obesity were 3.5% and 2.6%, and rates of heavy drinking were 18.4% and 5.4%, respectively.

### The presence of fatty liver is significantly associated with a higher BMI but not with alcohol intake or FIB-4 index

**Figure 2:**
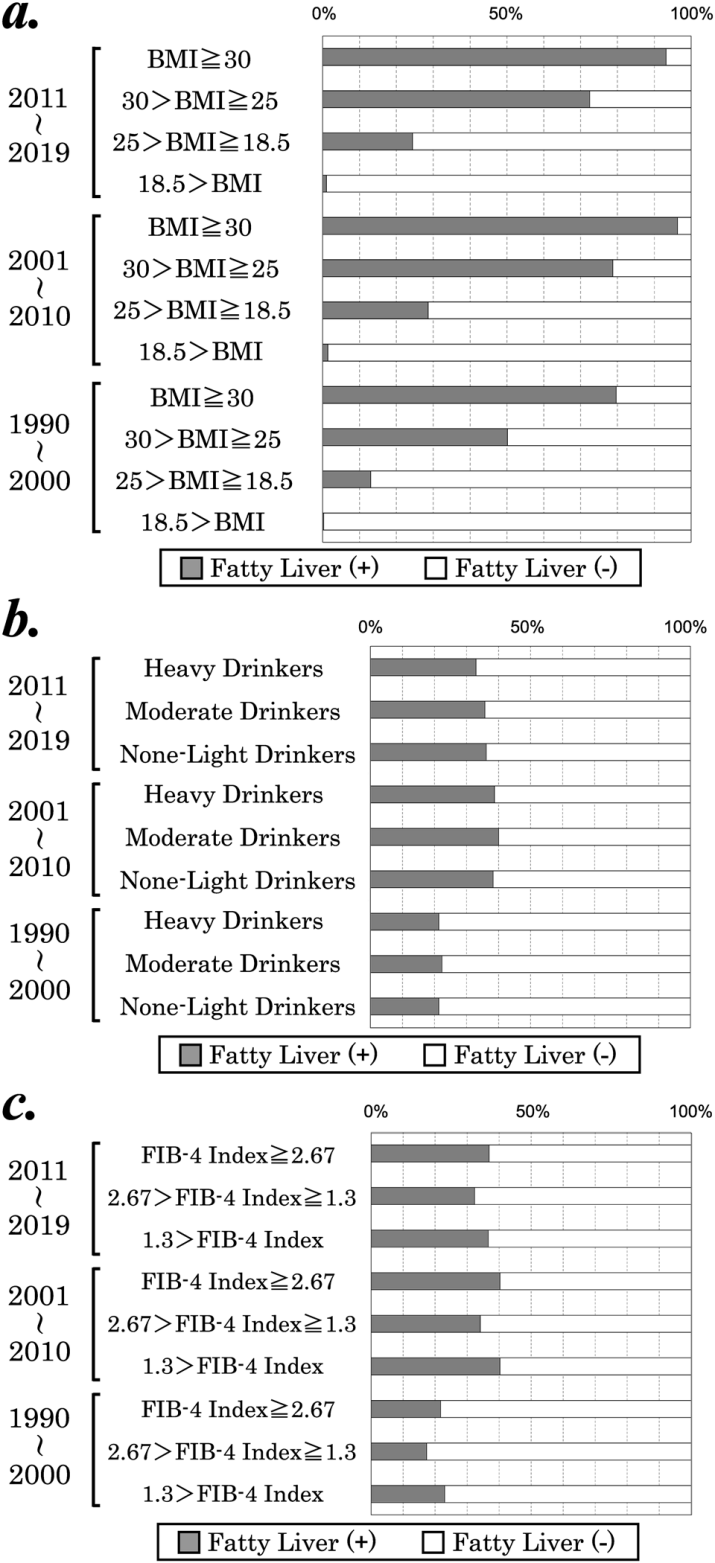
The prevalence of fatty liver in 1990–2000, 2001–2010, and 2011–2019 regarding body mass index (BMI) (**a**), alcohol intake (**b**), and fibrosis-4 (FIB-4) index (**c**).

Next, we evaluated the prevalence of fatty liver in all three periods by comparing the frequency distribution of BMI, alcohol intake, and the FIB-4 index (Fig. 2). As shown in Fig. 2a, an association between fatty liver and BMI was observed in all groups. However, the association of fatty liver with alcohol intake or the FIB-4 index could not be detected (Fig. 2b,c). Our results indicated that the presence of fatty liver significantly reflected BMI and obesity but not alcohol intake or FIB-4 index.

### FIB-4 index is a more useful indicator of chronic hepatitis development in comparison with fatty liver

To evaluate the predictive ability of FIB-4 index and fatty liver for chronic hepatitis or liver cirrhosis, we excluded 161,696 person-years of follow-up (48,407 participants) before April 2008 which did not have detailed information of comorbidities. We further excluded 209 person-years of follow-up (63 participants) which diagnosed with chronic hepatitis or liver cirrhosis at baseline. We further excluded 2,161 person-years of follow-up (441 participants) which diagnosed with liver, collagen, and hematological diseases.

**Figure 3:**
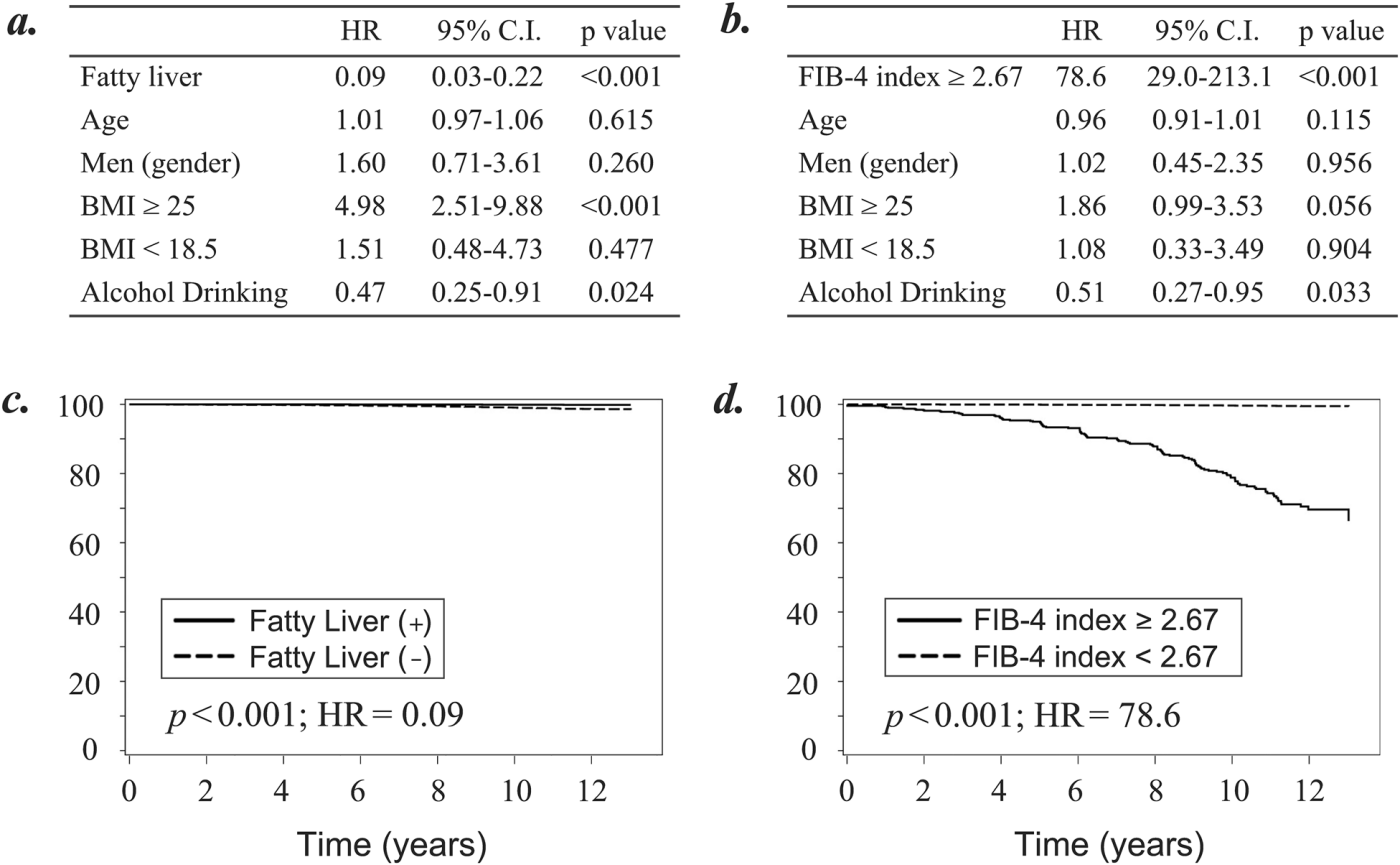
Cox regression analyses to evaluate the usefulness of fatty liver and FIB-4 index for prediction of chronic hepatitis development. (**a**) Cox regression analysis to evaluate chronic hepatitis development focusing on the presence of fatty liver. (**b**) Cox regression analysis to evaluate chronic hepatitis development focusing on the value of FIB-4 index. (**c**) Adjusted survival curves to evaluate the incidence of chronic hepatitis based on the Cox proportional hazards regression categorized by the presence of fatty liver. (**d**) Adjusted survival curves to evaluate the incidence of chronic hepatitis based on the Cox proportional hazards regression categorized by the value of FIB-4 index. All analyses were performed on the study participants after excluding those with chronic hepatitis, liver cirrhosis, and other diseases relating to liver, collagen, and hematological diseases at the time of entry. A *p*-value < 0.05 was considered statistically significant. *HR* hazard ratio; *C.I.* confidence interval.

Of the 252,000 subjects (61,857 participants), 114 developed chronic hepatitis. Two models of cox regression analyses were performed to identify the contributing factor focusing on fatty liver (Fig. 3a) or FIB-4 index (Fig. 3b) for development of chronic hepatitis. Furthermore, using the time-to-event data, the adjusted survival curves for Cox proportional hazards regression were plotted to evaluate the usefulness of fatty liver (Fig. 3c) or FIB-4 index (Fig. 3d) for predicting the risk of chronic hepatitis.

Unexpectedly, the risk of developing chronic hepatitis was higher in subjects without fatty liver than in those with fatty liver (Fig. 3a,c; Hazard ratio [HR] = 0.09; 95% confidence interval [CI], 0.03–0.22, *p* < 0.001). On the other hand, the risk of developing chronic hepatitis was much higher in subjects with a high FIB-4 index (≥ 2.67) than in those without it; (Fig. 3b,d; HR = 78.6; 95% CI, 29.0–213.1, *p* < 0.001). Assuming the situation with insufficient information of current or previous medical history, we performed the same analyses using all the 416,066 participants. Then, similar survival curves were plotted for chronic hepatitis development regarding fatty liver (Supplementary Fig. S1a; HR = 16.0, *p* < 0.001) and FIB-4 index (Supplementary Fig. S1b; HR = 58.6, *p* < 0.001).

As for other contributing factors, our data indicated that those who drink alcohol have the lower risk of developing chronic hepatitis (Fig. 3a,b; HR = 0.47 and HR = 0.51, *p* = 0.024 and *p* = 0.033).

### FIB-4 index is a more useful indicator of liver cirrhosis development in comparison with fatty liver

**Figure 4:**
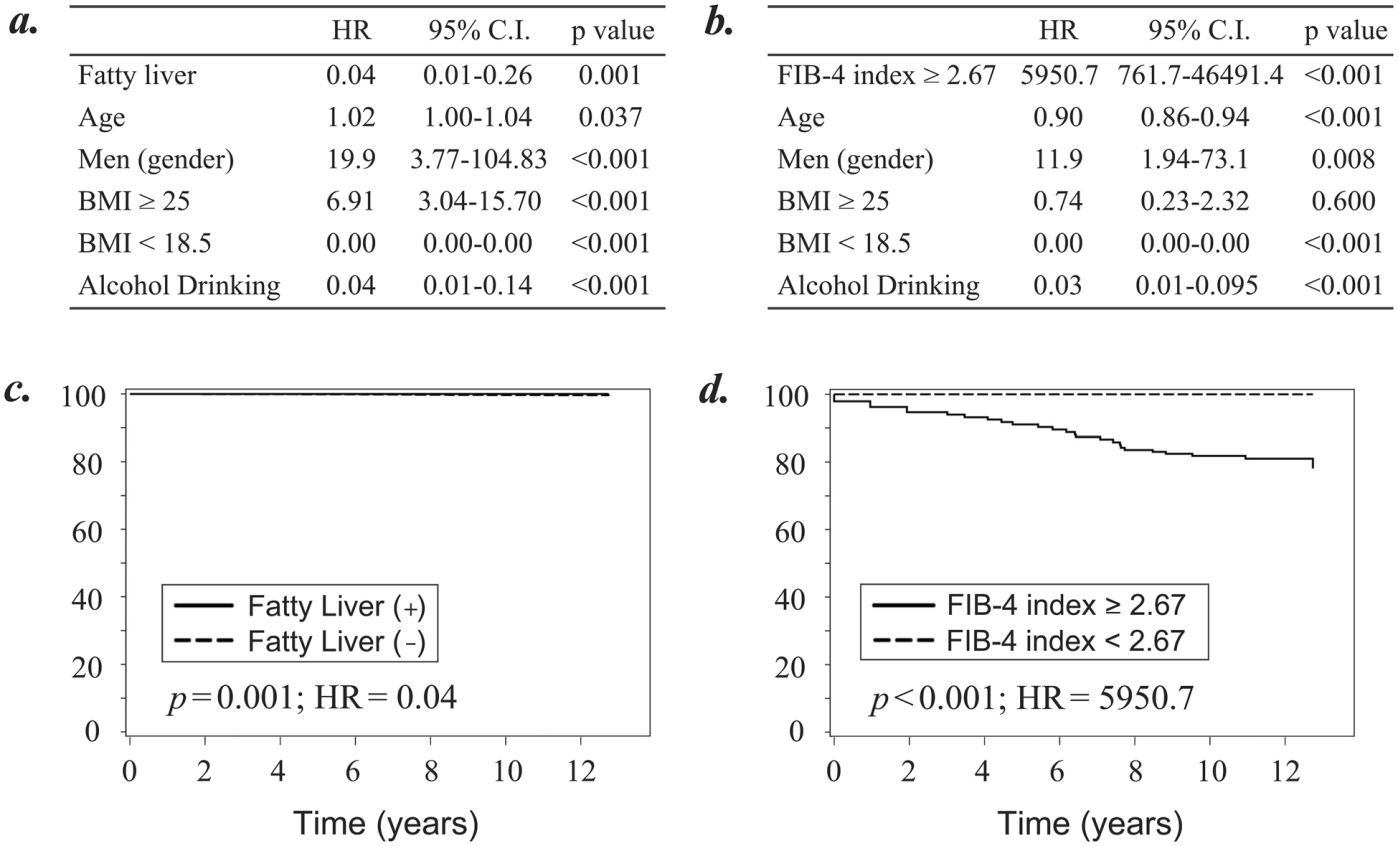
Cox regression analyses to evaluate the usefulness of fatty liver and FIB-4 index for prediction of liver cirrhosis development. (**a**) Cox regression analysis to evaluate liver cirrhosis development focusing on the presence of fatty liver. (**b**) Cox regression analysis to evaluate liver cirrhosis development focusing on the value of FIB-4 index. (**c**) Adjusted survival curves to evaluate the incidence of liver cirrhosis based on the Cox proportional hazards regression categorized by the presence of fatty liver. (**d**) Adjusted survival curves to evaluate the incidence of liver cirrhosis based on the Cox proportional hazards regression categorized by the value of FIB-4 index. All analyses were performed on the study participants after excluding those with liver cirrhosis, liver cirrhosis, and relating to liver, collagen, and hematological diseases at the time of entry. A *p*-value < 0.05 was considered statistically significant. *HR* hazard ratio; *C.I.* confidence interval.

Of the 252,000 subjects, 23 developed liver cirrhosis. Two models of cox regression analyses were performed to identify the contributing factor focusing on fatty liver (Fig. 3a) or FIB-4 index (Fig. 3b) for development of liver cirrhosis. Furthermore, using the time-to-event data, the adjusted survival curves for Cox proportional hazards regression were plotted to evaluate the usefulness of fatty liver (Fig. 4c) and the FIB-4 index (Fig. 4d) for predicting the risk of liver cirrhosis.

Similar to chronic hepatitis (Fig. 3), the risk of developing liver cirrhosis was higher in subjects without fatty liver than in those with fatty liver (Fig. 4a,c, HR = 0.04; 95% CI, 0.01–0.26, *p* = 0.001). The risk of developing liver cirrhosis was much higher in subjects with a high FIB-4 index (≥ 2.67) than in those without it (Fig. 4b,d; HR = 5950.7; 95% CI, 761.7–46,491.4, *p* < 0.001). Figure 4 showed that categorization based on the value of the FIB-4 index was more useful than categorization based on the presence of a fatty liver to predict development of liver cirrhosis. Assuming the situation with insufficient information of current or previous medical history, we performed the same analyses using all the 416,066 participants. Then, similar survival curves were plotted for chronic hepatitis development regarding fatty liver (Supplementary Fig. S2a; HR = 32.1, *p* < 0.001) and FIB-4 index (Supplementary Fig. S2b; HR = 532.4, *p* < 0.001).

As for other contributing factors, our data indicated that those who drink alcohol have the lower risk of developing liver cirrhosis (Fig. 3a,b; HR = 0.04 and HR = 0.03, *p* < 0.001 and *p* < 0.001).

## Discussion

In this large-scale longitudinal study, the prevalence of fatty liver in a generally healthy population greatly increased, whereas the rate of high FIB-4 index (≥ 2.67) did not markedly change during the 30 years of study. We further clarified the usefulness of a high FIB-4 index (≥ 2.67) in predicting the future development of chronic hepatitis or liver cirrhosis in a health checkup setting for the general population. It was suggested to be more useful than the diagnosis of fatty liver, as defined by ultrasonography, and was unaffected by the amount of alcohol consumed.

Liver diseases generally progress slowly, leading to hepatitis, cirrhosis, and cancer. In the past, viral hepatitis was the main cause, while presently, it has been attributed to fatty liver disease, including NAFLD and alcoholic liver disease^17^. NAFLD is a very common disease, and risk stratification for the development of advanced liver disease has been a challenge. Histological assessment is the leading method used to evaluate liver diseases, followed by non-invasive methods, including elastography using ultrasound and magnetic resonance imaging. However, these procedures require specific devices and are expensive to perform. The FIB-4 index has attracted attention as a risk stratification index from common laboratory tests, not only in patients with liver disorders but also in the general population.

We retrospectively analyzed the time trend of the FIB-4 index over 30 years and assessed how effectively the FIB-4 index predicted the development of chronic hepatitis and cirrhosis using a large-scale cohort of human health checkups. Interestingly, the FIB-4 index more efficiently predicted the future development of chronic hepatitis or cirrhosis in a generally healthy population than we expected. The risk stratification ability of the FIB-4 index was more accurate than the presence of fatty liver. It is quite meaningful to utilize the FIB-4 index as a risk predictor for the general population. Furthermore, the FIB-4 index consists of common and non-invasively determined values, including blood tests and patient age. The results obtained in this study have the potential to be widely generalized, including in settings with limited medical resources.

Interestingly, the FIB-4 index showed no apparent change over the 30 years. There have been no reports showing long-term changes in the FIB-4 index in the general population of Japan, and this finding is of great epidemiological importance. Given that the FIB-4 index, a possible predictor of advanced liver diseases, has remained unchanged, while the percentage of fatty liver disease has increased, the present results suggest alterations in the etiology of liver diseases in Japan, namely a decrease in viral hepatitis and an increase in non-viral etiologies, including fatty liver diseases^17^. Recent advances in the treatment of viral hepatitis are remarkable. The hepatitis C virus can be eradicated with direct-acting antivirals in almost all cases^18^, and the hepatitis B virus can be well controlled with nucleotide analogs^19,20^. However, the incidence of hepatocellular carcinoma in Japan has declined slightly in recent years^21^. In contrast, the proportion of non-viral etiologies of hepatocellular carcinoma and cirrhosis is increasing^17,22,23^. These results indicate that the diagnosis of viral hepatitis, which has been emphasized in the past, is becoming insufficient as a means of enclosing advanced liver diseases. The importance of the FIB-4 index, which can predict liver fibrosis and the future development of advanced liver diseases, might be greatly increasing.

Alcohol is known risk factor of liver disease progression. However, the meaning on fatty liver development is still controversial^1^. Indeed, our data in this study indicated that those who drink alcohol have the lower risk of developing chronic hepatitis or liver cirrhosis. However, the hazard ratios were relatively low. Furthermore, our data were based on participants of medical check-ups who have generally stronger health orientation, partially resulting in this reversal phenomenon. Considering these results, we could not conclude the effects of alcohol consumption from the results of this study.

Our study has several limitations. First, we defined chronic hepatitis and cirrhosis based on ultrasound findings. However, an ultrasound study could only detect relatively advanced chronic hepatitis and liver cirrhosis. As a result, patients with normal findings, namely no fatty liver, no chronic hepatitis, and no liver cirrhosis, might include patients who were in the process of progressing from fatty liver to burned-out nonalcoholic steatohepatitis^24^, which presents with no steatosis. Histological assessment by liver biopsy is the gold standard for identifying chronic hepatitis or liver cirrhosis; however, it was unrealistic and unsuitable for evaluation at the general population level, where the majority of subjects are at low risk for advanced liver disease. Second, some participants dropped out of the long-term follow-up because this study was the result of a voluntary medical examination. It is possible that the results might be underestimated, as those who developed diseases, including liver cancer or cirrhosis, during the study period were more likely to drop out.

In conclusion, from 1990 to 2019, the prevalence of fatty liver greatly increased, whereas the FIB-4 index and BMI did not change markedly in Japan. Categorization based on the FIB-4 index value is a much more useful indicator for the future development of chronic hepatitis and liver cirrhosis than the diagnosis of fatty liver.

## Methods

### Study subjects

A total of 423,358 asymptomatic subjects (93,935 participants) were enrolled in the health examination program for medical checkups from 1990 to 2019 at Kameda Medical Center Makuhari (Chiba, Japan). We excluded 6,629 person-years of follow-up data from 4,851 individuals because of missing or abnormal values for age, serum platelet count and alanine aminotransferase, height, weight, and alcohol habits. We also excluded 663 person-years of follow-up data from 363 individuals who did not undergo ultrasonography. Consequently, we retrospectively analyzed the medical records of 416,066 cumulative subjects (90,709 persons) (Fig. 1).

This study was approved by the ethics committee of the Kameda Medical Center (No. 17-075) and The University of Tokyo (No. 2865) and was performed in accordance with the ethical standards laid down in the 1964 Declaration of Helsinki and its later amendments. Written informed consent was obtained from all the study participants. All data were fully anonymized before the analysis.

### Diagnosis of fatty liver, chronic hepatitis, and liver cirrhosis

Abdominal ultrasonography was performed by well-trained ultrasonographers as previously reported^1^. All findings were first reported by an ultrasonographer and checked by another ultrasonographer, and finally confirmed and diagnosed by a medical doctor specializing in gastroenterology. When the diagnosis was difficult, the case was strictly evaluated in a conference consisting of approximately 20 gastroenterologists.

Fatty liver was diagnosed as previously reported^1^. A diagnosis of chronic hepatitis was comprehensively made with blunting of the hepatic limbus, coarse liver parenchyma, and elevated liver parenchymal echo level. A diagnosis of liver cirrhosis was also comprehensively made with irregular liver surface, coarse liver parenchyma, development of collateral vessels, atrophic right lobe and enlarged left or caudate lobe, and ascites.

In the analyses evaluating predictive abilities of FIB-4 index and fatty liver for chronic hepatitis or liver cirrhosis, we excluded patients with these ultrasound findings at baseline.

### Diagnosis of liver, collagen, and hematological diseases

Furthermore, patients with known existing diseases, which result in chronic hepatitis and cirrhosis, such as hepatitis B, hepatitis C, autoimmune hepatitis, Wilson disease, hemochromatosis, idiopathic portal hypertension, and chronic hepatitis with unknown etiology were excluded. Diagnosis was based on patient report at each medical check-up. We further excluded systemic lupus erythematosus, idiopathic thrombocytopenic purpura, and hematological malignancies (any leukemia, aplastic anemia, multiple myeloma, and myelodysplastic syndromes).

### Evaluation of FIB-4 index

The FIB-4 index was categorized into two levels (≥ 2.67 and < 2.67) or three levels (≥ 2.67, < 2.67, ≥ 1.30, and < 1.30), as previously reported^25,26^.

### Evaluation of alcohol consumption and BMI

The study subjects were classified into none-light drinkers (0–3 times per month), moderate drinkers (1–5 times per week), and heavy drinkers (6–7 times per week)^27^. For BMI, the participants were classified as underweight (< 18.5), normal weight (≥ 18.5 and < 25), overweight (≥ 25 and < 30), and obese (≥ 30) according to the World Health Organization classification^28^.

### Statistical analyses

We used multiple imputations to handle missing data under the assumption of “missing completely at random” because the data of the participants had missing values in drinking habits from January 1990 to March 2008. After we created a complete dataset for values other than drinking habits based on listwise deletion, we filled in missing values of drinking habits using a logistic regression imputation method. To impute missing values, we performed the fully conditional specification method^29^ by including variables potentially related to the missing data and variables correlated with liver conditions. We generated 10 imputed datasets using age (continuous data), sex (categorical data), BMI (continuous data), aspartate aminotransferase (continuous data), alanine aminotransferase (continuous data), platelet count (continuous data), serum high-density lipoprotein cholesterol (continuous data), serum low-density lipoprotein cholesterol (continuous data), serum triglycerides (continuous data), smoking habit (categorical data), and fatty liver diagnosed by echo-images (categorical data). For these imputed datasets, we selected one of the smallest quasi-likelihood under the independence model criterion values. We used datasets consisting of measured (observed) and predicted (imputed) data with missing values replaced by imputed values.

In multivariate analysis, we examined time trends in the risk of chronic hepatitis and liver cirrhosis using the FIB-4 index and fatty liver using recurrent-event survival analysis via Cox proportional hazards regression^30^ based on the counting process model^31^. This model assumed that the event for each individual did not depend on when or how many times it had occurred previously, including the beginning of observation, and assumed that the individual who had the event remained at risk as long as they did not drop out from the study. The incidence was estimated after considering all background characteristics. We used JMP 16.2, SAS 9.4 software (SAS Institute Inc. Cary, NC, USA), and Stata 17 (StataCorp, College Station, Texas, USA) for the statistical analyses.

## Supplementary Information

Supplementary Information.

## Data Availability

The datasets generated during and/or analyzed during the current study are not publicly available due to further uses for ongoing other clinical studies but are available from the corresponding author on reasonable request.
