# Supplementary material for: Fibrosis-4 index efficiently predicts chronic hepatitis and liver cirrhosis development based on a large-scale data of general population in Japan
Source: Sci Rep. 2022 Nov 27;12:20357. doi: 10.1038/s41598-022-24910-2 (PMC9701772; doi:10.1038/s41598-022-24910-2)
Supplement: Supplementary file 1 — Supplementary Information. [file 41598_2022_24910_MOESM1_ESM.pdf]

Table S1. Detailed data on the prevalence of fatty liver, fibrosis-4 (FIB-4) index, body mass index (BMI), and alcohol intake for the male subjects who were categorized into five age groups and three periods (1990–2000, 2001–2010, and 2011–2019).

Year	Age group (years)	N	Fatty liver		FIB-4 Index		BMI				Alcohol intake		
			present	absent	>=2.67	<2.67	<18.5	>=18.5, <25	>=25, <30	>=30	None-Light	Moderate	Heavy
1990-2000	<40	8417	2234 (26.5)	6183 (73.5)	5 (0.1)	8412 (99.9)	253 (3.0)	5832 (69.3)	2075 (24.7)	257 (3.1)	1976 (23.5)	5279 (62.7)	1162 (13.8)
	40-49	19331	5741 (29.7)	13590 (70.3)	64 (0.3)	19267 (99.7)	451 (2.3)	13271 (68.7)	5220 (27.0)	389 (2.0)	4741 (24.5)	11423 (59.1)	3167 (16.4)
	50-59	11521	3228 (28.0)	8293 (72.0)	199 (1.7)	11322 (98.3)	264 (2.3)	7901 (68.6)	3179 (27.6)	177 (1.5)	2814 (24.4)	6402 (55.6)	2305 (20.0)
	60-69	2899	741 (25.6)	2158 (74.4)	129 (4.4)	2770 (95.6)	98 (3.4)	2055 (70.9)	725 (25.0)	21 (0.7)	749 (25.8)	1484 (51.2)	666 (23.0)
	70＜	281	36 (12.8)	245 (87.2)	51 (18.1)	230 (81.9)	25 (8.9)	193 (68.7)	63 (22.4)	0 (0.0)	66 (23.5)	132 (47.0)	83 (29.5)
Total		42449 (0.0)	11980 (28.2)	30469 (71.8)	448 (1.1)	42001 (98.9)	1091 (2.6)	29252 (68.9)	11262 (26.5)	844 (2.0)	10346 (24.4)	24720 (58.2)	7383 (17.4)
2001-2010	<40	14455	5968 (41.3)	8487 (58.7)	4 (0.0)	14451 (100.0)	484 (3.3)	10037 (69.4)	3381 (23.4)	553 (3.8)	3856 (26.7)	8835 (61.1)	1764 (12.2)
	40-49	32088	16114 (50.2)	15974 (49.8)	52 (0.2)	32036 (99.8)	718 (2.2)	21243 (66.2)	8854 (27.6)	1273 (4.0)	8431 (26.3)	18692 (58.3)	4965 (15.5)
	50-59	33644	17625 (52.4)	16019 (47.6)	263 (0.8)	33381 (99.2)	705 (2.1)	22895 (68.1)	9272 (27.6)	772 (2.3)	8651 (25.7)	18749 (55.7)	6244 (18.6)
	60-69	9988	4562 (45.7)	5426 (54.3)	238 (2.4)	9750 (97.6)	289 (2.9)	7302 (73.1)	2281 (22.8)	116 (1.2)	2592 (26.0)	5273 (52.8)	2123 (21.3)
	70＜	1160	444 (38.3)	716 (61.7)	147 (12.7)	1013 (87.3)	81 (7.0)	826 (71.2)	237 (20.4)	16 (1.4)	330 (28.4)	554 (47.8)	276 (23.8)
Total		91335 (0.0)	44713 (49.0)	46622 (51.0)	704 (0.8)	90631 (99.2)	2277 (2.5)	62303 (68.2)	24025 (26.3)	2730 (3.0)	23860 (26.1)	52103 (57.0)	15372 (16.8)
2011-2019	<40	11810	3690 (31.2)	8120 (68.8)	16 (0.1)	11794 (99.9)	551 (4.7)	8290 (70.2)	2485 (21.0)	484 (4.1)	3417 (28.9)	7307 (61.9)	1086 (9.2)
	40-49	35845	15208 (42.4)	20637 (57.6)	89 (0.2)	35756 (99.8)	812 (2.3)	23589 (65.8)	9564 (26.7)	1880 (5.2)	9276 (25.9)	20546 (57.3)	6023 (16.8)
	50-59	42452	20108 (47.4)	22344 (52.6)	376 (0.9)	42076 (99.1)	851 (2.0)	27579 (65.0)	12038 (28.4)	1984 (4.7)	9348 (22.0)	23785 (56.0)	9319 (22.0)
	60-69	21906	10043 (45.8)	11863 (54.2)	594 (2.7)	21312 (97.3)	526 (2.4)	14863 (67.8)	5890 (26.9)	627 (2.9)	4828 (22.0)	11472 (52.4)	5606 (25.6)
	70＜	4666	1741 (37.3)	2925 (62.7)	549 (11.8)	4117 (88.2)	174 (3.7)	3304 (70.8)	1086 (23.3)	102 (2.2)	1347 (28.9)	2147 (46.0)	1172 (25.1)
Total		116679 (0.0)	50790 (43.5)	65889 (56.5)	1624 (1.4)	115055 (98.6)	2914 (2.5)	77625 (66.5)	31063 (26.6)	5077 (4.4)	28216 (24.2)	65257 (55.9)	23206 (19.9)

Table S2. Detailed data on the prevalence of fatty liver, fibrosis-4 (FIB-4) index, body mass index (BMI), and alcohol intake for the female subjects who were categorized into five age groups and three periods (1990–2000, 2001–2010, and 2011–2019).

Year	Age group (years)	N	Fatty liver		FIB-4 Index		BMI				Alcohol intake		
			present	absent	>=2.67	<2.67	<18.5	>=18.5, <25	>=25, <30	>=30	None-Light	Moderate	Heavy
1990-2000	<40	4751	219 (4.6)	4532 (95.4)	4 (0.1)	4747 (99.9)	583 (12.3)	3711 (78.1)	387 (8.1)	70 (1.5)	2378 (50.1)	2165 (45.6)	208 (4.4)
	40-49	11628	1077 (9.3)	10551 (90.7)	13 (0.1)	11615 (99.9)	695 (6.0)	9201 (79.1)	1517 (13.0)	215 (1.8)	6300 (54.2)	4813 (41.4)	515 (4.4)
	50-59	6089	911 (15.0)	5178 (85.0)	64 (1.1)	6025 (98.9)	259 (4.3)	4766 (78.3)	954 (15.7)	110 (1.8)	3486 (57.3)	2287 (37.6)	316 (5.2)
	60-69	1290	278 (21.6)	1012 (78.4)	57 (4.4)	1233 (95.6)	61 (4.7)	927 (71.9)	275 (21.3)	27 (2.1)	745 (57.8)	460 (35.7)	85 (6.6)
	70＜	158	43 (27.2)	115 (72.8)	31 (19.6)	127 (80.4)	12 (7.6)	96 (60.8)	49 (31.0)	1 (0.6)	103 (65.2)	49 (31.0)	6 (3.8)
Total		23916 (0.0)	2528 (10.6)	21388 (89.4)	169 (0.7)	23747 (99.3)	1610 (6.7)	18701 (78.2)	3182 (13.3)	423 (1.8)	13012 (54.4)	9774 (40.9)	1130 (4.7)
2001-2010	<40	11045	1382 (12.5)	9663 (87.5)	2 (0.0)	11043 (100.0)	2234 (20.2)	7776 (70.4)	839 (7.6)	196 (1.8)	5705 (51.7)	4922 (44.6)	418 (3.8)
	40-49	22386	4613 (20.6)	17773 (79.4)	15 (0.1)	22371 (99.9)	2580 (11.5)	16612 (74.2)	2572 (11.5)	622 (2.8)	12052 (53.8)	9320 (41.6)	1014 (4.5)
	50-59	19834	6380 (32.2)	13454 (67.8)	77 (0.4)	19757 (99.6)	1613 (8.1)	14830 (74.8)	2986 (15.1)	405 (2.0)	11667 (58.8)	7177 (36.2)	990 (5.0)
	60-69	4671	1544 (33.1)	3127 (66.9)	61 (1.3)	4610 (98.7)	373 (8.0)	3553 (76.1)	663 (14.2)	82 (1.8)	3035 (65.0)	1436 (30.7)	200 (4.3)
	70＜	590	166 (28.1)	424 (71.9)	50 (8.5)	540 (91.5)	42 (7.1)	457 (77.5)	79 (13.4)	12 (2.0)	392 (66.4)	157 (26.6)	41 (6.9)
Total		58526 (0.0)	14085 (24.1)	44441 (75.9)	205 (0.4)	58321 (99.6)	6842 (11.7)	43228 (73.9)	7139 (12.2)	1317 (2.3)	32851 (56.1)	23012 (39.3)	2663 (4.6)
2011-2019	<40	9319	925 (9.9)	8394 (90.1)	2 (0.0)	9317 (100.0)	1923 (20.6)	6346 (68.1)	807 (8.7)	243 (2.6)	4603 (49.4)	4278 (45.9)	438 (4.7)
	40-49	28432	5242 (18.4)	23190 (81.6)	22 (0.1)	28410 (99.9)	4104 (14.4)	19888 (69.9)	3511 (12.3)	929 (3.3)	14015 (49.3)	12403 (43.6)	2014 (7.1)
	50-59	29696	8929 (30.1)	20767 (69.9)	158 (0.5)	29538 (99.5)	3433 (11.6)	20869 (70.3)	4420 (14.9)	974 (3.3)	15693 (52.8)	12000 (40.4)	2003 (6.7)
	60-69	13220	4344 (32.9)	8876 (67.1)	253 (1.9)	12967 (98.1)	1498 (11.3)	9369 (70.9)	2043 (15.5)	310 (2.3)	8274 (62.6)	4277 (32.4)	669 (5.1)
	70＜	2494	705 (28.3)	1789 (71.7)	237 (9.5)	2257 (90.5)	311 (12.5)	1769 (70.9)	373 (15.0)	41 (1.6)	1797 (72.1)	608 (24.4)	89 (3.6)
Total		83161 (0.0)	20145 (24.2)	63016 (75.8)	672 (0.8)	82489 (99.2)	11269 (13.6)	58241 (70.0)	11154 (13.4)	2497 (3.0)	44382 (53.4)	33566 (40.4)	5213 (6.3)

Figure S1. Adjusted survival curves based on the Cox proportional hazard model to evaluate the incidence of chronic hepatitis using the data of all the 416,066 participants.

a) Adjusted survival curves to evaluate the incidence of chronic hepatitis categorized by the presence of fatty liver. b) Adjusted survival curves to evaluate the incidence of chronic hepatitis categorized by the value of FIB-4 index.

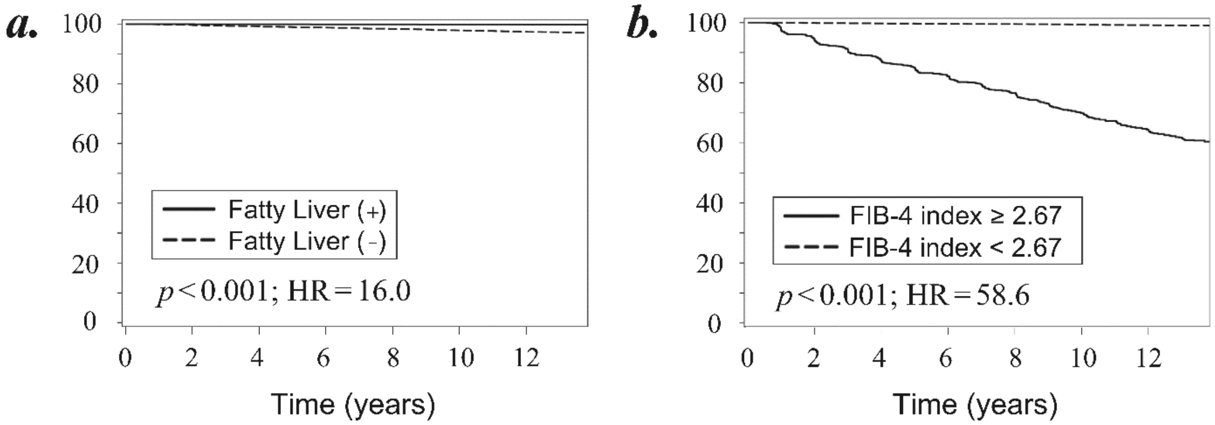

A p-value <0.05 was considered statistically significant. HR, hazard ratio

Figure S2. Adjusted survival curves based on the Cox proportional hazard model to evaluate the incidence of liver cirrhosis using the data of all the 416,066 participants.

a) Adjusted survival curves to evaluate the incidence of liver cirrhosis categorized by the presence of fatty liver. b) Adjusted survival curves to evaluate the incidence of liver cirrhosis categorized by the value of FIB-4 index.

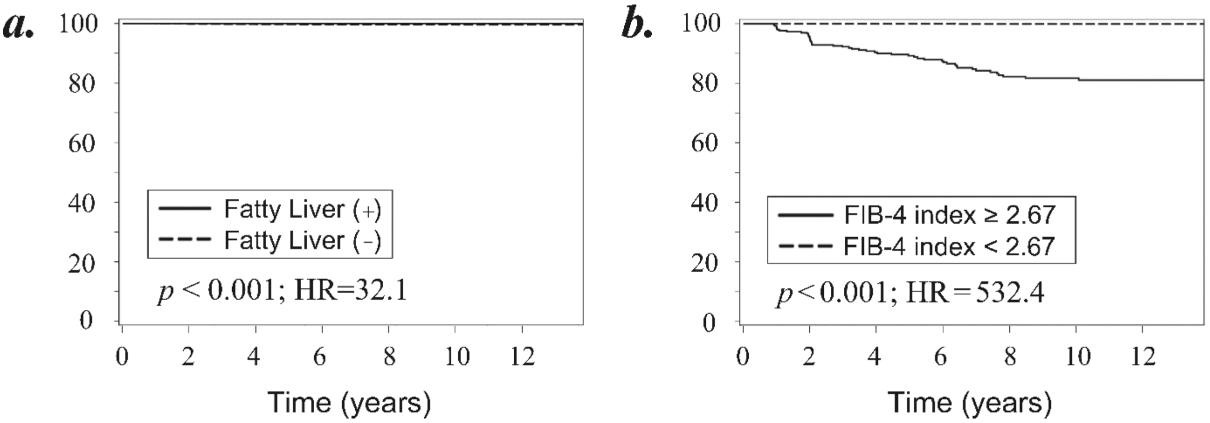

A p-value <0.05 was considered statistically significant. HR, hazard ratio
